# Friends’ Closeness and Intimacy From Adolescence to Adulthood: Art Captures Implicit Relational Representations in Joint Drawing: A Longitudinal Study

**DOI:** 10.3389/fpsyg.2020.573140

**Published:** 2020-10-22

**Authors:** Sharon Snir, Tami Gavron, Yael Maor, Naama Haim, Ruth Sharabany

**Affiliations:** ^1^ Department of Art Therapy, Tel-Hai College, Tel Hai, Israel; ^2^ School of Creative Arts Therapies, University of Haifa, Haifa, Israel; ^3^ School of Psychology Science, University of Haifa, Haifa, Israel; ^4^ Academic College of Tel-Aviv Jaffa, Tel Aviv, Israel

**Keywords:** joint drawing, intimate friendship, closeness, internal representations, longitudinal study

## Abstract

This longitudinal study, employing a mixed-methods explanatory design, explored the power of art to express aspects of one’s inner world using the joint drawing technique, which allows for observation and treatment of implicit representations of relationships. At Time 1 (T1, 1977–1978), 200 adolescents created a joint drawing with either a good friend or with a classmate who was not a friend and filled out the Intimate Friendship Scale (IFS) in relation to their best friend. In 2014 (T2), 36 women and 21 men from the original cohort completed the IFS with regard to a good friend and with regard to their spouse. The drawings were analyzed qualitatively to define pictorial phenomena that may be indicative of closeness. The analysis was conducted in accordance with the phenomenological approach to art therapy and with the principles of thematic analysis. Fourteen pictorial phenomena were defined, and a scale was constructed to quantitatively evaluate the extent to which each phenomenon was present in the joint drawing. This yielded a closeness score for each drawing. Quantitatively, no correlations were found between intimacy as measured by IFS at T1 and at T2. In contrast, there was a correlation between the degree of closeness in the joint drawing at T1, and the IFS score with the partner in T2, suggesting continuity over the 36-year time span. This correlation was likewise found when examined separately among participants who drew with a friend. The multivariate ANOVA (MANOVA) results showed a marginally significant effect for the interaction between closeness in drawing and drawing with a friend/non-friend – on IFS. An ANOVA showed that the IFS regarding the participant’s best friend and their romantic partner at T2 was higher when the closeness in the drawing at T1 was higher. There was also a significant interaction between closeness in the drawings and the participant’s IFS score regarding their best friend at T1. The differences between the joint drawing with the close friend and the non-friend are discussed. These findings, from a span of over 36 years, thus contribute to the validity of the IFS and the joint drawing technique when assessing closeness and intimacy.

## Introduction

One of the central qualities of the art produced in the process of art therapy is its remarkable ability to express aspects of the artist’s inner world (Robbins, 2001). This quality contributes to the use of art as a way for clients to explore themselves and as a tool through which the therapist is able to observe and assess developmental and transformative processes in the course of treatment (Betts, 2012). In this mixed-method longitudinal study, spanning 36–37 years and following participants from adolescence to adulthood, we investigated this quality of art, focusing on its ability to express representations of closeness and intimacy.

### Intimate Friendship in Adolescence and Adulthood

The development of intimacy in close-knit relationships is described by developmental psychologists as one of the main tasks of adolescence (Sullivan, 1953; Erikson, 1994; Gilmore and Meersand, 2014). According to Sharabany’s definition of intimate friendship, to successfully accomplish this task, teens need to establish relationships characterized by mutual closeness and trust with their peers. Within such relationships, adolescents feel free to be honest, spontaneous, and open with their friends. Intimate friendships involve a deep familiarity between the two sides, including an awareness of the friend’s feelings, preferences, and beliefs, as well as knowledge of details about their personal life. Such friends enjoy spending time together; in fact, they prefer to spend most of their time together exclusively with one another, without other peers. When they are apart, on the other hand, they tend to feel the absence of the friend in an acute manner (Sharabany, 1974, 1994a). The experience of intimacy in adolescence has also been found to be correlated with healthy psycho-social functioning (Chou, 2000; Rubin et al., 2004; Selfhout et al., 2009; Van Harmelen et al., 2016, 2017) and is a key contributor to developing healthy romantic relationships in later adolescence and in adulthood (Connolly and Goldberg, 1999; Scharf and Mayseless, 2001).

According to theoreticians and researchers, intimate relationships form a foundation of social support and contribute to healthy emotional, social, and personality development not only in adolescence, but in later years, and throughout a person’s life (Leone and Hawkins, 2006; Sneed et al., 2012; Carmichael et al., 2015; Waldinger and Schulz, 2016; Layman et al., 2019). The nature of the intimacy created within these relationships changes throughout the various stages of life, with each age period giving rise to different worries, needs, and stress factors that affect the intimate interactions characteristic of that particular phase (Sharabany et al., 1981, 2008; Sharabany, 1994a; Prager, 1997; Eshel et al., 1998). Intimate friendship in adulthood occupies a different niche than in adolescence. Several studies show that the introduction of romantic relationships affects partners’ other intimate friendships. Compared to single people and non-parents, intimate friendship among married couples and parents is lower (Eshel et al., 1998). Moreover, depending on the attachment style of the individual, adult friendship shifts toward one’s romantic partner at the expense of intimate friendships (Mayseless et al., 1997). Many studies document the different functions of adult friendship for men and women. While women’s friendships are based on self-disclosure as a central feature, men tend to base their friendships on common activities (e.g., Reis et al., 1985).

Close-knit relationships stem from the dynamics between individual people, dynamics which develop over time, and in response to a variety of different circumstances, and thus aspects of a close-knit relationship that were significant at one point in the relationship’s evolution may not necessarily be significant at later stages (Leone and Hawkins, 2006). Furthermore, various life events, as well as gender, age, family status, and social status, may affect the way an individual selects friends and establishes friendships (Pahl and Pevalin, 2005). Therefore, the nature of such relationships and the kind of intimacy involved may change in the course of a person’s life according to their particular circumstances. Nevertheless, research shows that people’s internal representations of relationships, as expressed in interpersonal relationships established by the individual, are somewhat consistent throughout their lives (Sharabany, 1994a; Chopik et al., 2014; Stern et al., 2018).

Internal representations of interpersonal relationships are based on memories of interactions with significant others that are aggregated into units of information and shaped by the person’s inherent individual attributes, such as gender, temperament, and so on (Brazelton and Cramer, 1991; Bernstein, 2004; Orbach, 2009). These representations constitute an internal working model (Bowlby, 1969), which functions as a kind of script for the individual, bridging between past experiences and current behavior and helping them decipher and adapt to the situation by organizing, structuring, interpreting, and shaping the way they perceive themselves and others (Beebe and Lachmann, 1988; Beebe et al., 1997). Internal representations have an explicit layer that is conscious, overt, and subject to verbal expression (Rudy and Grusec, 2006), as well as an implicit layer, which is unconscious, covert, and non-verbal (Stern, 2004; Araneda et al., 2010; Greenwald and Banaji, 2017). The implicit components consist of procedural information processing principles, behavioral strategies, and physiological regulation mechanisms (Fraley, 2002; Crittenden, 2006; Jones, et al., 2018), which in the course of an individual’s life manifest themselves as feelings, emotions, and behaviors, while remaining less conscious, less accessible to verbal expression (Fonagy, 2001; St Quinton and Brunton, 2017; Gavron and Mayseless, 2018), less conscious, and less manageable.

Although these two layers, the implicit and the explicit, are simultaneously present in every single interaction with the other (Greenwald and Banaji, 2017), various approaches have identified implicit communication as the key to understanding interpersonal relationships (Solan, 1991; Kiesler, 1996; Pally, 2005; Crittenden 2006). Nevertheless, the implicit and non-verbal aspects of interpersonal relationships are inherently difficult to study, and the research literature suggests that despite the importance of implicit communication in relationships, studies on the subject of intimacy in adolescence have thus far been conducted *via* the explicit means of self-reporting (Sharabany et al., 1981; Ferguson et al., 2018; Van Petegem et al., 2018). An art-based approach, which provides a look into the artist’s inner world, including its implicit aspects, may therefore have an important contribution to make to the research on representations of interpersonal relationships, including representations of closeness and intimacy, and their consistency throughout a person’s life, from adolescence into adulthood.

### Joint Drawing as an Assessment

The use of art as an assessment is based on the notion that the work produced by the artist constitutes a projective space, wherein internal content can be externalized (Machover, 1949; Rubin, 1999). Pictorial phenomena may therefore reveal fragments of the artist’s inner world (Frank et al., 1994; Gilory et al., 2012). One of the common techniques used for the purposes of assessment in art therapy is the joint drawing, wherein two people share a single piece of paper to make a drawing. In the field of art therapy, the subject of joint drawing has been widely used as a tool to examine family relationships (Bing, 1970; Landgarten, 2013), relationships among siblings (Rehmann, 1979), among parents and children (Proulx, 2003; Gavron and Mayseless, 2015, 2018; Regev and Snir, 2017), among couples (Snir and Wiseman, 2016), among friends (Sharabany and Hertz-Lazarowitz, 1981; Sharabany et al., 1994; Sharabany, 1994b), among clients paired up with one another in group settings (Barker and Brunk, 1991), and among therapists drawing on a single page together with their clients (Silverman, 2013; Furneaux-Blick, 2019).

In each of these configurations, the joint drawing constitutes an invitation for the partners to interact on the page through color, movement, and shape (Snir and Hazut, 2012; Gavron, 2013). The joint activity of making the drawing allows the partners to express their representation of past relationships and to recreate situations that are typical of the partners’ relationship (Snir and Hazut, 2012). Joint drawing is a task that is new to most participants, and as such, it invites them to express implicit content that is nonconscious and hard to express verbally; therefore, the result provides a much wider and deeper insight into their psyche than that provided by verbal diagnostic tools (Gennar and Tamanza, 2014; Gavron and Mayseless, 2015; Snir and Wiseman, 2016). Various researchers have identified the joint drawing as an expression of non-verbal communication and pointed out how the collaborators’ perceptions of themselves, the other, their relationship, and recurring patterns of communication manifest themselves in this shared space (Sharabany and Hertz-Lazarowitz, 1981; Sharabany et al., 1994; Gavron, 2013; Regev and Snir, 2017).

According to the phenomenological approach to art therapy (Betensky, 1995; Guttmann and Regev, 2004; Hazut, 2014), in the context of joint drawing, the assessment process is based on the observation of pictorial phenomena, which manifest themselves both in the course of making the drawing and in the final product, and expresses the artists’ experience as well as their inner world. Proponents of this approach as a research and assessment tool maintain that these assessments are based on the study of perceivable and definable elements, which leaves little room for projection-based interpretation (Somer and Somer, 1997). Another advantage of this approach is that the definitions of pictorial phenomena and behaviors it employs, enable one to examine their correlations with external criteria, while applying the procedures of empirical research (Gavron and Mayseless, 2015; Snir and Wiseman, 2016). Hence, the present study has chosen to rely on the principles of the phenomenological approach in analyzing joint drawings, while focusing on their ability to express closeness and intimacy in relationships. The choice of this particular subject matter is based on previous studies in which researchers showed closeness and intimacy to be the central attributes of a relationship expressed through the joint drawing process (Sharabany and Hertz-Lazarowitz, 1981; Molad, 1991; Snir and Hazut, 2012; Gavron and Mayseless, 2018). According to these studies, closeness and intimacy manifest themselves in joint drawings through a variety of phenomena, such as pictorial continuity, use of shared or parallel elements, stylistic similarities between the two artists, proximity between the two artists on the page in a manner that does not create conflict or defacement, moderate contact between the two artists, completion and connection of one artist’s elements with the other’s, the presence of friendly images, and the absence of aggressive images (Molad, 1991; Snir and Hazut, 2012; Gavron, 2013).

## The Present Study

The present study examined the correlation between expressions of closeness in joint drawings made in adolescence (some by pairs of adolescents who identified as close friends, and some by pairs who did not define themselves as friends) and intimacy in friendships, both in adolescence and in adulthood, as well as intimacy in romantic relationships in adulthood. We examined intimacy *via* explicit, declarative means, namely, a self-reporting questionnaire – the Intimate Friendship Scale (IFS; Sharabany, 1974, 1994b). The drawings, meanwhile, allowed for a wider assessment of closeness, including both explicit and implicit components of the relationships. Throughout the research, we asked what pictorial phenomena are indicative of closeness in joint drawings made by pairs of adolescent friends and classmates. We likewise wished to examine whether there was a correlation to be found between intimacy, as assessed and measured based on pictorial phenomena, and declared intimacy, as assessed and measured by way of the self-reporting questionnaire. An additional focus of our investigation was the question of whether closeness and intimacy remained consistent over the years, with the passage from adolescence into adulthood.

## Methodology

The present research, which aims to study the evaluative attributes of joint drawings, is a longitudinal study based on data collection performed at two points in time, 36–37 years apart. It employs a mixed-methods explanatory design strategy (Creswell et al., 2003), which combines qualitative analysis of joint drawings with quantitative data collected *via* self-reporting questionnaires. This study is part of a larger research dealing with intimacy in close relationships (Sharabany, 1978; Herz-Lazarowitz et al., 1983; Vagman, 2014; Lev-Eshel, 2018; Chen, 2019; Maor, 2019).

### Participants

The participants at the first data collection point (T1, 1977–1978) were 200 adolescents (born between 1960 and 1963) studying in grades 9 and 11 at two high schools in Northern Israel (Haifa). Whole classes were invited to participate in the study, and only a few individuals chose to abstain. Out of these, 107 also filled in questionnaires as adults at the second data collection point (T2, 2014). However, some of the data from T1 had unfortunately been lost, and thus ultimately the study consisted of 57 participants – 36 women and 21 men, who filled in the questionnaires at both collection points. The age of participants at T1 ranged between 14 and 17 (*M* = 15.71, *SD* = 0.99), and between 51 and 54 (*M* = 52.33*, SD* = 1.28) at T2. The participants who were located as adults and who agreed to participate in the second stage of the study did not differ in terms of intimacy levels in friendship, as measured in adolescence, from those who could not be located or did not agree to participate in T2 (*t*_(397)_ = −2.20 ns). Also, no demographic disparities were found between the two groups. The sample attributes are described in Table 1.

**Table 1:** Sample distribution by demographic variables.

		Women *n* = 36		Men *n* = 21	
		*N*	%	*n*	%
Grade at T1	9th	20	55.6	15	71.4
	11th	16	44.4	6	28.6
Mother’s education	8 years or less	3	8.3	1	4.8
	8–12 years	11	30.6	6	28.6
	Technical	15	41.7	4	19
	Academic	5	13.9	8	38.1
	Not specified	2	5.6	2	9.5
Father’s education	8 years or less	4	11.1	1	4.8
	8–12 years	11	30.6	6	28.6
	Technical	4	11.1	3	14.3
	Academic	15	41.7	9	42.9
	Not specified	2	5.6	2	9.5
Relationship status at T2	Married	21	58.3	15	71.4
	In a relationship	2	5.6	2	9.5
	Divorced	10	27.8	3	14.3
	Single	1	2.8	1	4.8
	Not specified	2	5.6	-	-

### Measurements

#### Sociometric Questionnaire (T1)

The adolescents were asked to specify the names of six same-gender friends with whom they “hang out,” as well as the name of their best friend. This information was used to classify friends and non-friends.

#### Relational Intimacy Questionnaire (T1 and T2)

Relational intimacy was measured in both rounds of research by way of the IFS questionnaire (Sharabany, 1974, 1994b; Sharabany et al., 1981). The questionnaire consists of 32 items, in which participants rate the extent to which each statement describes their relationship (1 = “This sentence does not describe my relationship at all” and 6 = “This sentence describes my relationship very well”). The 32 items cover eight dimensions, with four items composing each subscale: (1) frankness and spontaneity (e.g., “I feel free to talk to him/her about almost everything”); (2) sensitivity and knowing (e.g., “I know how he/she feels about things without him/her telling me”); (3) attachment (e.g., “I like him/her”); (4) exclusiveness (e.g., “It bothers me to have other people around when the two of us are doing something together”); (5) giving and sharing (e.g., “If he/she wants something I let him/her have it even if I want it too”); (6) imposition (e.g., “I can use his/her things without asking permission”); (7) common activities (e.g., “I like to do things with him/her”); and (8) trust and loyalty (e.g., “I defend him/her if needed”). Since the sample size was small, and in view of the large number of sub-scales, for the present study, we decided to use only the overall mean score on this scale, omitting the sub-scales, in order to reduce the number of statistical tests.

Participants filling in the questionnaire in the first round of research (T1) were asked to think of their best friend and give their answers based on their relationship with him/her using the IFS. In the second round of research (T2), participants were asked to fill in the IFS questionnaire three times: once for their best friend, once for a romantic partner, and once for a close family member. The present study utilized the first two: best friend and romantic partner. A higher score on this scale indicates greater intimacy. The questionnaire has content validity, discriminative validity, and criterion validity (Sharabany, 1994b). The total score reliability coefficient (Cronbach’s alpha) was found to be high in many studies in various countries (Chou, 2000; Mikulincer and Florian, 2000; Shechtman et al., 2002; Oliva and Arranz, 2005). In the current study, the scale’s internal consistency reliabilities of the overall score at T1 were *α* = 0.94, 0.84, and 0.95, for the general sample, and for male and female, respectively. At T2, Cronbach’s alpha was 0.95 for all of these three groups.

### Procedure (T1)

The first round of data collection (1977–1978) was composed of three stages.

#### Stage A: Classroom Questionnaire

The IFS questionnaire (Sharabany, 1974) was administered collectively in the participating classrooms, without a teacher present, by a research assistant who read the questions out loud to the students. Three additional research assistants were present in the classroom to answer any questions that might arise. At the end of the process, the participants were told that some of them would be invited to take part in the next stage of the study in pairs. Since it was not possible to invite everybody, the researchers selected pairs of friends and of adolescents who were not friends, based on their sociometric declarations, at random.

#### Stage B: Selecting Pairs for Joint Drawing

Based on the results of the sociometric questionnaire, the participants were divided into three groups of pairs with varying degrees of reciprocity (reciprocal – both of the participants had included each other in their list of six; partially reciprocal – both of the participants had included each other, but not at the same rank on the list; non-reciprocal – only one of the two participants had included the other). Due to the sample size, this classification resulted in a final categorization of “friends” (reciprocal and partially reciprocal) and “not friends” (non-reciprocal).

#### Stage C: Making a Joint Drawing

Around 2–10 weeks after the completion of the questionnaires, the pairs were invited at random order to present themselves in a separate classroom, where they were asked by a research assistant to perform a number of tasks, one of which was the joint drawing task (Sharabany, 1978; Sharabany and Hertz-Lazarowitz, 1981). The category of friends-non friends as well as degree of reciprocity of friendship within the pair was unknown to the research assistant. The behavior of the pairs while drawing was observed and written down in detail on corresponding coding forms; this information, which detailed who drew which part in the joint drawing, assisted the drawing analysis phase.

Seated side by side, the pair were given an A3 size page and two sets of coloring materials. At one end of the page, there was a box with six colored markers: red, blue, yellow, green, brown, and black. At the other end, there was a box of colored pencils in the same exact colors. The placement of the two boxes was randomly switched from one pair to the next. The partners were instructed to make a drawing together (on the same page) and were given 12 min to accomplish this task.

### Procedure (T2)

The second data collection round took place 36–37 years later (2014). Hundred and sixty of the participants in T1 were located *via* phone inquiries and online means, and 107 of them expressed willingness to continue their participation in the study. Once their consent was obtained, participants were sent a link to a set of online questionnaires, one of which was the IFS intimacy questionnaire utilized in the present study.

### Ethical Aspects

The invitation to participate in the study was extended to the entire classroom. In the first round of data collection, both the students and their parents were given the option to refuse to participate in the study. Participants in the second round of data collection signed an additional consent form, as adults. The study was certified as ethical by the Chief Scientist at the Israeli Ministry of Education and received the approval of the Ethics Commission at the University of Haifa.

## Part 1: Qualitative Analysis and Building a Tool for the Assessment of Intimacy in Joint Drawings

### Drawing Analysis Procedure

The analysis of the drawings was performed jointly by the authors of the present article, a team which consisted of an experienced researcher and practitioner in the fields of clinical and developmental psychology (Sharabany), two graduate students in clinical psychology (Maor and Haim), and two art therapists specialized in the study of joint drawings (Snir and Gavron). The purpose of this content analysis was to define the phenomena occurring in the drawings that might give some indication about the relationship between the artists. Based on the procedures of the phenomenological approach to art therapy (Betensky, 1995; Guttmann and Regev, 2004; Hazut, 2014) and taking into account, the results of joint drawing analyses we have conducted in past studies (Sharabany et al., 1994; Snir and Hazut, 2012; Gavron and Mayseless, 2015; Regev and Snir, 2017), we paid attention to the depicted images, their position on the page, and the way they were drawn (for instance, the amount of pressure applied through the drawing utensil or the continuity of the line). These are all phenomena that can be observed, described, and agreed upon (Betensky, 1995), and therefore, their definition constitutes a critical stage in research aspiring toward minimizing projection-based interpretation (Somer and Somer, 1997). In addition to relying on the results of previous studies and clinical instructions, we also strived to define, throughout this process, any potentially significant pictorial phenomena observed in the course of analysis, and as such, our analysis can be defined as inductive (Wall et al., 2013). Unlike the analysis procedure we employed in previous studies, which relied among other things on our observation of the creative process, in the present study, the analysis was ultimately conducted mainly through the investigation of the final product of the joint interaction. Additionally, we referred to the notes taken by the experimenters during the drawing process in order to understand which elements were drawn by which individual. The analysis was performed in the stages prescribed by the thematic analysis method (Braun and Clarke, 2006), which has been used as a means of processing visual artistic information in the past (Fraser and al Sayah, 2011; Chilton and Scotti, 2014). In the first phase of the thematic analysis method, becoming familiar with our primary data, we spread the drawings out on a table and looked at them while conducting a discussion and sharing notes about the different ways the adolescents reacted to the instructions and drew together. In the second stage of generating initial codes, which took place in an additional meeting, we defined, with no prior system of categorization, the pictorial phenomena that we perceived as significant toward understanding the relationship between the two artists of the drawing. These pictorial phenomena include use of similar colors by both artists or drawing two separate drawings side by side on the same page. In the third stage of defining themes, which took place in the same meeting as stage two, we defined the list of pictorial phenomena that emerged during the coding process. The fourth and fifth stages took place in a third meeting and involved reviewing the themes and defining/naming them. Each phenomenon was defined as a continuous five-point scale upon which the relationship between the partners can be defined, in a way that would be relevant to all couples in a comprehensive manner without creating data overload.

### Drawing Analysis Findings

**Figure 1:**
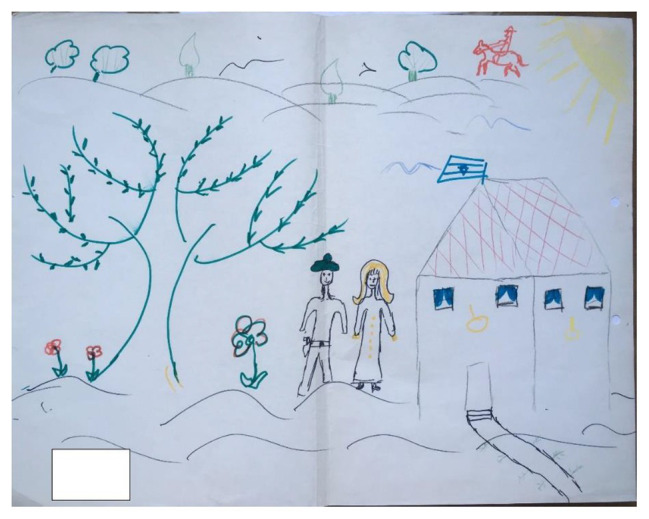
Example of a drawing exhibiting a high degree of closeness based on the analysis of the drawing’s content and pictorial elements.

**Figure 2:**
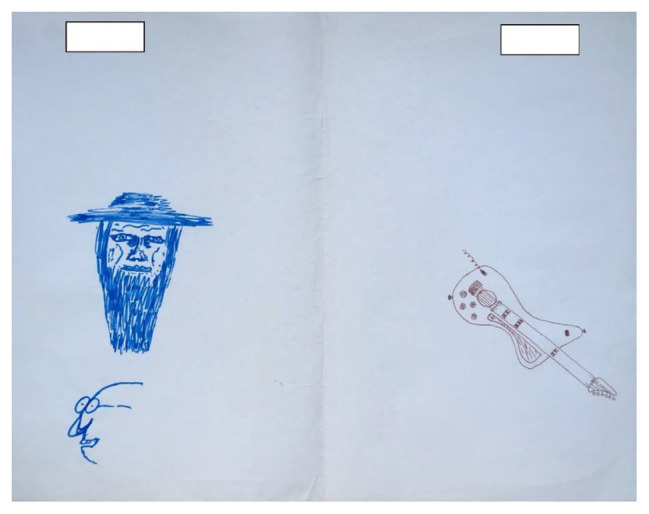
Example of a drawing where the artists drew two separate pictures on the page.

The qualitative analysis of the drawings defined 14 pictorial phenomena indicative of the relationship between the artists. The first and most significant among them refers to the shared drawing space and determines whether the work consists of one cohesive drawing in a shared space (such as in Figure 1), or whether it consists of two drawings drawn side by side on the same page (such as in Figure 2). This phenomenon was defined both as a scale between cohesive and separate, and as a dichotomy which divided the drawings into two groups (using a median): one cohesive drawing or two separate drawings side by side on a page. Each of these groups had pictorial phenomena that were applicable to it.

#### Phenomena Applicable to Cohesive Drawings: Where the Two Participants Produced a Single Integrated Drawing

**Figure 3:**
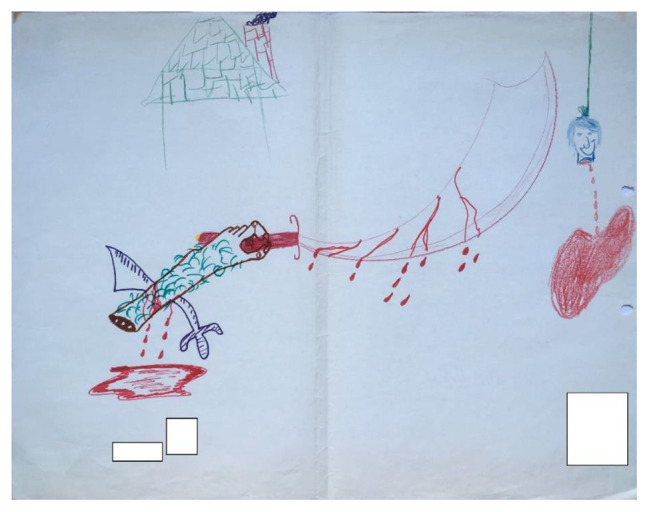
Example of a drawing exhibiting a low degree of closeness based on the analysis of the drawing’s content and pictorial elements.

**Figure 4:**
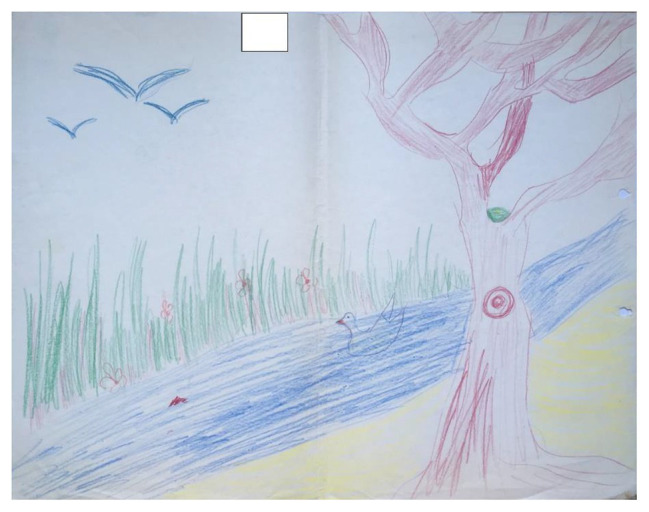
Example of a drawing where the artists drew a single cohesive picture.

1. Drawing space: this relates to how much room on the page the cohesive drawing takes up. In comparing Figure 1 with Figure 3, we can see that the drawing in Figure 1 is spread over the entire page and takes up a lot of space, whereas in Figure 3, the drawing takes up relatively small areas on the page.
2. Distinctness: this relates to whether the contributions of each artists are distinct from the other, or whether the drawing consists of a single cohesive image/narrative (symbiosis). Figure 1, for example, despite being a cohesive drawing, contains distinct elements that were drawn by each of the artists separately, such as the house which was drawn by one partner and the tree which was drawn by the other, whereas Figure 4 consists of one central image, with few elements that can be attributed to any one single artist.

#### Phenomena Applicable to Separate Drawings: Where the Two Participants Produced Two Separate Drawings on the Same Page

1. Distance from center: this describes the difference between the distances of the two artists’ drawings from the middle of the page. In Figure 2, the two images – the guitar on the right side and the faces on the left side – are positioned at a similar distance from the center line; however, there were drawings where one artist’s drawing was closer to the middle than the other, and there was a difference in their positioning in relation to the middle of the page.
2. Distance from each other: this describes how far apart the two drawings are from each other, and conversely how close they are to the edge of the page. In Figure 2, the two artists maintained a relatively large amount of space between their two drawings (the guitar and the faces).
3. Shared materials: this describes whether the two artists used the same drawing implements or whether each part of the drawing was drawn using different materials. For example, in Figure 2, we can see that the two artists used markers.
4. Subject matter similarity: this looks at whether the two separate drawings have similar subject matter or whether they have two disparate subject matters. In Figure 2, for example, there is no discernable link between the depicted subject matters. On the right we see the image of a musical instrument, while on the left we see images of human faces.
5. Formal similarity: this looks at whether there are formal elements common to the two parts of the drawing.
6. Color similarity: this is a scale indicating the degree of similarity in the colors used for the two parts of the drawing. For instance, in Figure 3, despite the violent and disturbing subject matter, we can see both formal and color similarities between the two parts of the drawing. The shingles on the roof drawn in green pencil are similar both in color and shape to the hair drawn on the arm in marker by the second artist, which may constitute an unconscious attempt to create closeness in spite of expressions of aggressiveness. The red heart on the right side of the drawing is also similar in color and shape to the puddle of blood on the left side of the drawing.
7. Orientation: this looks at whether the figurative elements in each part are turned inward toward the middle of the page or outward toward the edge of the page. We can see in Figure 2 that the blue face is turned away from the other artist, in the outward direction.

#### Phenomena Applicable to Both Types of Drawings

1. Paired images: this scale evaluates the presence of images that come in pairs within the drawing. Figure 1, for example, is rich in paired images: the flowers to the right of the tree, the two human characters, and even the two central branches of the tree.
2. Aggressiveness: this examines the presence of aggressive images in the drawing. The blood and the decapitated head in Figure 3 are both examples of aggressive images.
3. Friendly elements: this evaluates the presence of friendly images (smiles, outreached hands, words of love, flowers, hearts, etc.), such as, for example, the smile on the face of the human figure on the right in Figure 1.
4. Difference in size: this compares the size of the elements or parts drawn by each artist, while examining how much room each element takes up on the page. In Figure 2, it appears that the two artists maintained relative parity in terms of the space each took up on the page.

For each of the phenomena defined above, we established a scale from 1 to 5, with the aim of evaluating to what extent each of these phenomena appear in the joint drawing. Next, the joint drawings were rated on the scales by authors N. Haim and Y. Maor, independently. The inter-rater reliability between the two authors was calculated using Cohen’s kappa test, and the values for the different subscales (see Tables 2 and 3) ranged between 0.56 and 0.84 (*M* = 0.73).

**Table 2:** Phenomena included in the closeness measure for joint drawings that consisted of one cohesive drawing.

Pictorial phenomenon and kappa coefficient	Values				
	1	2	3	4	5
Aggressiveness *k* = 0.65	Death and violence	A predatory animal	Threats and warnings	Something broken, competition, a crack	No aggressiveness
Friendly elements *k* = 0.74	Neutral – no friendly elements	One element	Two or three elements	Four elements	The entire drawing seems friendly

**Table 3:** Phenomena included in the closeness measure for joint drawings that consisted of two separate drawings on the same page.

Pictorial phenomenon and kappa coefficient	Values				
	1	2	3	4	5
Distance from the middle *k* = 0.87	Significant difference in each part’s distance from the middle	2	3	4	The parts are equidistant from the middle
Distance from each other *k* = 0.70	Each part is at the opposite edge of the page	2	3	4	The two parts are right next to each other
Subject matter similarity *k* = 0.68	Zero similarity in subject matter	Little similarity	Some similarity	A lot of similarity	Almost identical subject matter
Formal similarity *k* = 0.66	No common formal elements	One common element	Two common elements	Three common elements	Four or more common elements
Color similarity *k* = 0.71	No color similarity between the two parts	Low degree of similarity	Medium degree of similarity	High degree of similarity	The two parts use the exact same colors
Orientation *k* = 0.84	Both parts face outward	One part is neutral the other faces outward	One part faces outward, the other inward/both are neutral	One part is neutral, the other faces inward	Both parts face inward
Paired images *k* = 0.56	No paired images	One paired image	Two or three paired images	Four paired images	Five or more paired images
Friendly elements *k* = 0.87	Neutral – no friendly elements	One element	Two or three elements	Four elements	The entire drawing seems friendly

For each of the two kinds of joint drawing – one cohesive drawing or two separate drawings on the same page – we calculated the closeness exhibited in the drawing based on the scores recorded for the relevant phenomena. After calculating Cronbach’s alpha coefficient for each scale, some of the phenomena were removed from the measure due to insufficient reliability. We found internal consistency coefficients of 0.39 for the remaining scales proper to cohesive drawings (“aggressiveness” and “friendly elements”) and 0.64 for the remaining scales proper to separate drawings (“distance from the middle,” “distance from each other,” “formal similarity,” “color similarity,” “orientation,” “paired images,” “friendly elements,” and “subject matter similarity”). The scales for the final list of phenomena, after removing those found to be not reliable, are presented in Table 2 for cohesive drawings and in Table 3 for separate drawings. Based on this analysis, each participant was accorded a score for closeness in the joint drawing (average of the scores recorded for the relevant scales of the measure, based on whether they drew one cohesive drawing or two separate drawings with their partner).

## Part 2: Quantitative Analysis Examining the Correlation Between Degree of Closeness in the Joint Drawings, and Intimacy with Best Friend in Adolescence, as Well as Intimacy with Best Friend and with Romantic Partner in Adulthood

In the second part of the study, we examined the correlation between the degree of intimacy in relationships as measured using the self-reporting questionnaires in T1 and T2 and the degree of closeness as measured in the joint drawings. The study’s hypotheses were that:

1. A positive correlation would be found between the degree of closeness in the joint drawings and the degree of the individual’s intimacy in relationships with their best friend in adolescence, as well as intimacy with their best friend and with their romantic partner in adulthood.
2. This correlation would be different for pairings of friends as opposed to pairings of adolescents who were not friends.

### Preliminary Intimacy Analysis

No correlation was found between intimacy in friendship during adolescence and intimacy in friendship or in romantic relationships in adulthood, but there was a significant positive correlation (*r* = 0.76, *p* < 0.002) between intimacy in friendship and intimacy in romantic relationships in adulthood. In comparing between men and women in terms of levels of intimacy, it was found that in adolescence, the degree of intimacy in friendship was higher among teenage girls [*t*(55) = −3.11, *p* < 0.001]. The full results of the analysis are aggregated in Table 4.

**Table 4:** Averages of Intimate Friendship Scales in adolescence and in adulthood, comparison between men and women.

	Male		Female		Total						
Intimacy scale	*M*	*SD*	*M*	*SD*	*M*	*SD*	Std. error	Mean difference	*T*	*p*	*Df*
Best friend in adolescence (T1)	4.49	0.39	4.96	0.61	4.79	0.58	0.14	−0.46	−3.11	0.03	55
Best friend in adulthood (T2)	4.37	0.82	4.67	0.77	4.56	0.79	0.21	−0.30	−1.39	0.17	55
Romantic partner (T2)	4.81	1.11	4.95	0.83	4.9	0.94	0.26	−0.13	−0.52	4.81	53

### Correlation Between Closeness in the Joint Drawings and Intimacy

The study’s first hypothesis, according to which a positive correlation exists between the degree of closeness in the joint drawings and the degree of intimacy in the individual’s relationship with their best friend in adolescence, and with their best friend and their romantic partner in adulthood, was examined using the Pearson’s test.

In accordance with this hypothesis, a significant positive correlation was found between the degree of closeness observed in the joint drawings and the degree of intimacy with a romantic partner in adulthood (*r* = 0.30, *p* < 0.05). However, no correlation was found between closeness in the joint drawings and intimacy in friendship, either in adolescence or in adulthood.

In accordance with the study’s second hypothesis, when this correlation was examined separately for pairings of friends and non-friends, the findings showed that among friends (*n* = 46), the degree of closeness in the joint drawings was correlated with intimacy with a romantic partner in adulthood (*r* = 0.31, *p* < 0.05), while among participants who were paired up with non-friends (*n* = 10), the degree of closeness in the joint drawings, examined using the Pearson’s test, was not correlated with any of the three intimacy scores.

In order to investigate the effect produced by the degree of closeness in drawing, the friendship variable, and the interaction between them on the intimacy variables, while taking into account the existing link between the dependent variables and their common variance, we conducted a multivariate ANOVA (MANOVA). The closeness in drawing variable was converted into a dichotomous variable using the median, while the two independent variables were closeness in drawing and friendship among the drawing partners. The dependent variables were intimacy in friendship in adolescence, intimacy in friendship in adulthood, and intimacy in romantic relationships in adulthood.

**Figure 5:**
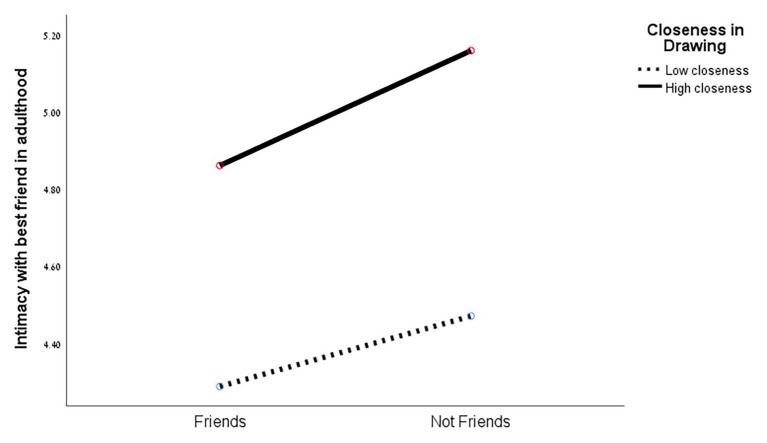
Intimacy with best friend in adulthood and closeness in drawing (in adolescence).

**Figure 6:**
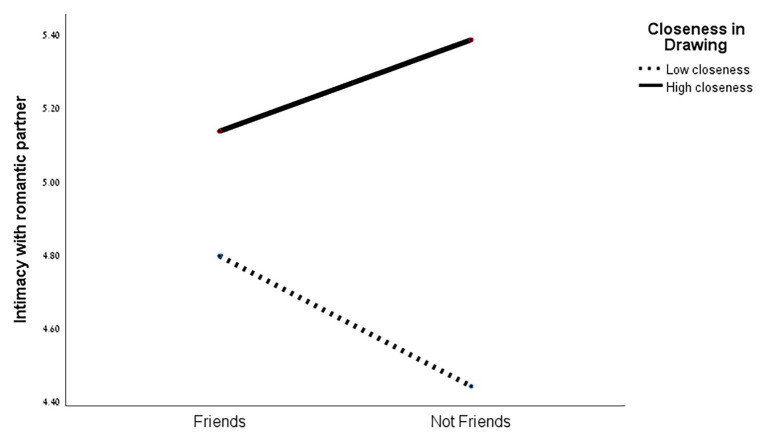
Intimacy with romantic partner in adulthood and closeness in drawing (in adolescence).

The MANOVA results showed no significant effect of closeness in drawing, or of friendship, on the intimacy scales. However, a marginally significant effect was found for the interaction between both variables [*F*(3,48) = 2.36, *p* < 0.083]. At the ANOVA level, a significant difference was found between the two levels of closeness in drawing for both intimacy measures in adulthood: with the best friend [*F*(1,50) = 5.36, *p* < 0.025], and with the romantic partner [*F*(1,50) = 3.57, *p* < 0.065]. As can be seen in Figures 5, 6, intimacy with the best friend and with the romantic partner in adulthood was higher when closeness in drawing was higher.

**Figure 7:**
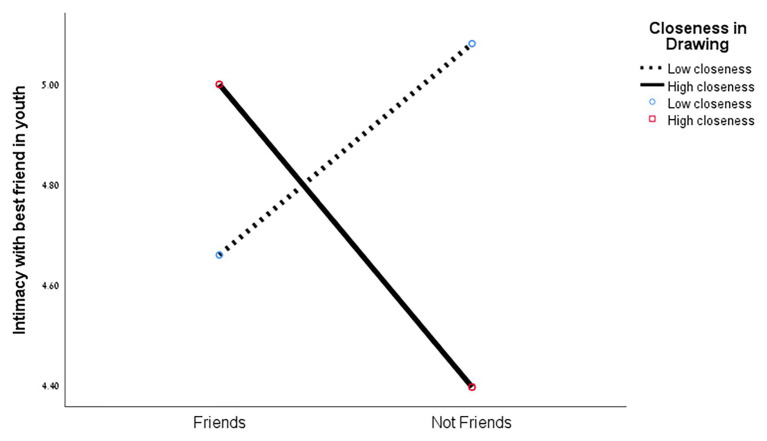
Intimacy with best friend in adolescence and closeness in drawing.

The interaction between friendship and closeness also had a significant effect on intimacy with the best friend in adolescence [*F*(1,50) = 6.15, *p* < 0.017]. As can be seen in Figure 7, among friends, when closeness in the drawing was low, intimacy with best friend in adolescence was lower (*M* = 4.65, *SD* = 0.11) than when closeness in the drawing was high (*M* = 4.99, *SD* = 0.15). Conversely, among non-friends, when closeness in the drawing was low, intimacy with best friend in adolescence was higher (*M* = 5.05, *SD* = 0.23) than when closeness in the drawing was high (*M* = 4.39, *SD* = 0.28).

## Discussion

The present longitudinal study investigated the correlations between the degree of closeness indicated by joint drawings produced in adolescence, intimacy in friendship – both in adolescence and in adulthood, and intimacy in romantic relationships in adulthood. Two rounds of data collection were conducted 36–37 years apart. The study also focused on the question of continuity in the levels of closeness and intimacy over the years, in the transition from adolescence to adulthood. The study supports the validity of the tool of joint drawings as a way of looking at closeness and intimacy, and examines the possibility of change in one’s capacity for intimacy in close relationships through the evolution of the intimate friendship variable over time.

The study’s hypotheses were that there would be a positive correlation between the degree of closeness observed in the joint drawings and intimacy in close relationships in both adolescence and adulthood, and that this correlation would be different for drawing partners who were friends, as opposed to partners who were not friends. The hypotheses were partially confirmed.

### Pictorial Phenomena Representing Closeness in the Joint Drawings

In the qualitative portion of the study, we defined pictorial phenomena, as observed in the joint drawings, which may attest to the degree of closeness experienced by the drawing partners. In the absence of thorough documentation of the joint drawing process and unlike previous studies in which we examined joint drawings (Snir and Hazut, 2012; Gavron and Mayseless, 2015, 2018; Yakovson and Snir, 2019), our analysis relied mainly on the final product. Nevertheless, we were able to define 14 pictorial phenomena, 10 out of which were found to be effective in assessing closeness in the drawings. The phenomena, which were defined inductively by way of the thematic analysis method, were similar in nature to the pictorial phenomena found and defined in the contexts of free parent/child joint drawings (Yakovson and Snir, 2019), structured parent/child paintings (Gavron, 2013), and joint drawings made by romantic partners (Snir and Hazut, 2012). Therefore, pictorial phenomena, such as distance between the marks left by each artist on the page, friendly or aggressive images, and stylistic similarity (subject matter, color, and form), were reaffirmed by the present study as acting in the capacity of “pictorial words” that speak of closeness and intimacy in the different contexts of pair drawing.

### Closeness in the Joint Drawings and Intimacy

The assessment of the extent to which each pictorial phenomenon manifested itself in the drawings formed the basis for building a quantitative index to assess the existence of closeness in the joint drawings. In accordance with the first hypothesis, the degree of closeness in the joint drawings was found to be positively correlated with intimacy with a romantic partner in adulthood. The higher the degree of closeness exhibited in the joint drawing made by the individual in adolescence, the higher was the level of intimacy in their relationship with their romantic partner in adulthood. Similarly, the level of intimacy with a best friend in adulthood was higher among individuals who, as adolescents, had made a joint drawing that was classified as exhibiting a high degree of closeness, as opposed to those whose joint drawing was classified as exhibiting a low degree of closeness. The findings indicate that adolescents whose drawings exhibited pictorial markers of closeness (a cohesive collaborative drawing including more friendly and less aggressive elements; and/or two separate drawings with a smaller distance between them; and/or those with more similarities in terms of subject matter, color, and shapes), reported a higher level of intimacy with their best friend and with their romantic partner in adulthood, as compared with adolescents whose drawings exhibited less pictorial markers of closeness. These findings corroborate those from previous studies that established a correlation between expressions of closeness in joint drawings – such as physical proximity on the page, stylistic similarities, and the presence of predominantly friendly images as opposed to aggressive images – and assessment of various aspects of relationships through self-reporting questionnaires, thus supporting the validity of the joint drawing as a means of assessing relationships (Gavron, 2013; Snir and Wiseman, 2016).

Curiously, no similar correlation was found among the adolescents in the concurrently assessed degree of closeness based on the joint drawings and the intimacy questionnaire scores. Nevertheless, when it came to our second research hypothesis, it was found that the relation between closeness in drawings and intimacy in friendship in adolescence was not the same among drawing partners who were friends as among partners who were not friends. Among pairings of friends, the pattern we saw was similar to the one hypothesized, as well as to the correlation found when the dependent variable was intimacy in adulthood. Namely, when the degree of closeness in the joint drawing was high, the level of intimacy with the friend referenced in the questionnaire was higher than when the degree of closeness in the joint drawing was low. Among drawing partners who were not friends, however, the situation was reversed: it was individuals with high scores for closeness in the joint drawings who reported lower levels of intimacy with their best friends.

In light of this finding, we can hypothesize that adolescents with greater intimacy in their relationships with their best friends tended to draw in a more reserved, less collaborative, and more distant manner when asked to participate in a joint task with a classmate with whom they did not have close relations. On the other hand, adolescents who found it difficult to create intimacy in their relationships with close friends used the joint drawing task as an opportunity to connect and create closeness with a classmate who was not their friend. The use of art-making as a way of creating change, or expressing a desired circumstance, a dream, or a wish, has been written about by theoreticians (Cavallo and Robbins, 1980; Storr, 1991), recognized in clinical practice (Maclagan, 2005), and supported by preliminary research (Snir and Wiseman, 2016). The element of “wishes” in relationships is also a central component in the assessment of internal working models, perceived by many theoreticians to include representations of one’s self, the other, and one’s wishes for the relationship (Luborsky and Crits-Christoph, 1998). In other words, this finding may be explainable by the therapeutic quality of the joint drawing as an opportunity to create a desired interaction, one that differs from the explicit interactions the individual usually experiences in their existing relationships. These qualities are at the basis of the widespread use of joint drawings as a therapeutic tool and as a means of stimulating development and change, alongside their use as expressions of the artists’ representations and perceptions of relationships for assessment and diagnostic purposes (Gavron and Mayseless, 2018).

### Intimacy Over Time

One of the central contributions of the present research is its observations regarding the correlation of variables measured 36–37 years apart. The findings show that there is a link between information about closeness gleaned from the joint drawing task completed in adolescence and intimacy in adulthood, and that expressions of closeness in a joint drawing made with a friend in adolescence were correlated with self-reported assessment of intimacy with a friend and with a romantic partner in adulthood. Accordingly, a correlation was also found between intimacy with a peer at adolescence as was measured by a self-report questionnaire, and intimacy in a relationship with a partner in adulthood, as measured using the same questionnaire. These findings are in line with the vast body of knowledge attesting to the interconnectedness between relationships with friends in adolescence and the development of romantic intimacy in adulthood (Sharabany, 1994a; Zimmer-Gembeck et al., 2001; Crockett and Randall, 2006; Furman and Shomaker, 2008; Allen et al., 2020). The link between the drawings in adolescence and the self-reported assessment in adulthood, across different periods of the individuals’ development, attests to the validity of the two measures. This fact emphasizes the importance of assessing intimacy also through artistic means, as a window through which we can gain a glimpse into expressions of the individual’s internal relational representations. In addition, the findings support the hypothesis that there is continuity in the individual’s close relationship from adolescence to adulthood, and this continuity is manifested in the individual’s representations of close relationships (Snir and Wiseman, 2016; Gavron and Mayseless, 2018).

Interestingly, no correlation was found between intimacy with a friend in adolescence and intimacy with a friend in adulthood, as measured by the self-report questionnaires. It is possible that this finding is related to changes that occur over time in the nature of relationships with friends. Theoreticians and researchers suggest that in adulthood, when people establish a romantic partnership and/or marriage, the number and quality of friendships changes (Kearns and Leonard, 2004); attachment and other emotional and supportive functions that were fulfilled by friends are more directed toward one’s romantic partner (Meeus et al., 2007).

### Limitations of the Present Study

The long period of time over which the study took place provided us with an extraordinary research opportunity. Having said that, the fact that the study was not initially planned to be longitudinal, brought with it several limitations. First, the analysis of the joint drawings produced in the first phase of the research was performed long after they had been drawn, by researchers who were not present at the time of their making. As we know, information gleaned from observing the drawing process has been proven significant by previous research (Snir and Hazut, 2012; Gavron, 2013). Therefore, our ability to accurately assess closeness based on the drawings was limited. Likewise, assessing closeness in drawings that consisted of one cohesive picture proved to be more problematic in this study, with quite a few phenomena having to be omitted from the final measure due to insufficient reliability, resulting in only two pictorial phenomena proper to these kinds of drawings that ended up being factored into the closeness score. Here too, the fact that the analysis of the drawings was conducted by judges who had not been present during the process of their making minimized our ability to make any conclusions about closeness in these drawings (and, in this sense, weakened the odds of finding a correlation with the intimacy questionnaires in adulthood, which was ultimately found despite this drawback). In addition, the participants’ experience of the drawing task, which usually constitutes an inseparable component of understanding the drawings, had not been documented and was therefore not available to us at the analysis stage.

The size of the sample constituted another limitation. Individuals who had dropped out of the study over the years, drawings that had been lost and the division into sub-groups, lowered the number of participants significantly. Future research examining the correlation between intimacy in relationships and expressions of closeness in drawings in various contexts over time that will document the drawing process, the participants experience of the drawing task, and increase the sample size will be able to corroborate this study’s findings, as well as investigate gender differences, which were not examined in the scope of the present study. Another possible research direction would be to observe series of joint drawings made in the context of a romantic partnership and their ability to predict intimacy in the relationship over time. Similarly, it would be interesting to research the possible correlation between aspects of joint drawings made by parent/child pairings, and the children’s ability to develop intimacy in relationships as adults, as well as the ability to evaluate the efficacy of dyadic art therapy on the ability to stimulate change through joint drawings.

## Data Availability Statement

The raw data supporting the conclusions of this article will be made available by the authors, without undue reservation.

## Ethics Statement

The studies involving human participants were reviewed and approved by Chief Scientist at the Israeli Ministry of Education and Ethics Commission at the University of Haifa. Written informed consent to participate in this study was provided by the participants’ legal guardian/next of kin.

## Author Contributions

The present study is part of a larger researched planned and executed by RS. YM led the data analysis process and wrote research reports as a part of her Master’s thesis, under the direction of RS. All of the authors participated in preparing the assessment scales, with YM and NH taking charge of the analysis. TG and SS wrote the article based on the Master’s thesis. All authors contributed to the article and approved the submitted version.

### Conflict of Interest

The authors declare that the research was conducted in the absence of any commercial or financial relationships that could be construed as a potential conflict of interest.

The reviewer RL declared a shared affiliation with several of the authors, TG, YM, NH, and RS, to the handling editor at time of review.
